# Lynch syndrome caused by SINE-VNTR-Alu-F retrotransposon insert in *MSH6* confirmed after 20 years of testing: a case report and literature review

**DOI:** 10.1186/s13053-025-00324-9

**Published:** 2025-10-14

**Authors:** Wenche Sjursen, Eva Kathrine Svaasand, Bodil Gilde, Anuradha Ravi, Katinka Madtzog Korseth, Ashish Kumar Singh, Jostein Johansen, Olaug Kristin Rødningen, Sofie Geck Sevatdal, Siv Anita Hegre, Maren Fridtjofsen Olsen, Kristine Misund

**Affiliations:** 1 Department of Medical Genetics, St Olavs University Hospital, Trondheim, Norway; 2 Department of Clinical and Molecular Medicine, Norwegian University of Science and Technology, Trondheim, Norway; 3 Department of Medical Genetics, Oslo University Hospital, Oslo, Norway

**Keywords:** Lynch syndrome, Retrotransposons, Whole genome sequencing, Long-read sequencing

## Abstract

**Background:**

Lynch syndrome is due to error in DNA mismatch repair (MMR) genes caused by germline pathogenic variants. For some families highly suspicious of Lynch syndrome, the diagnosis may not be confirmed.

**Case presentation:**

We present a family where Lynch syndrome has been suspected for 20 years. Although haplotyping and tumor analyses suggested Lynch syndrome, newer sequencing methods such as whole-genome sequencing and long-read sequencing, were needed to detect the underlying genetic cause of their cancer predisposition. We identified a > 3kbp retrotransposon (RT) insertion in *MSH6* to be the causative germline variant. Further, we reviewed the literature for RT events in Lynch syndrome families and found a total of 40 RT cases, making up about 0.5% of Lynch cases. Two-third of the RTs were shorter ALU-elements (< 500 bp).

**Conclusions:**

Although RTs insertions do not seem to be a common cause of Lynch syndrome, the number might be underestimated because of the difficulties in detecting these variants with well-established methods like Sanger sequencing and NGS target sequencing.

**Supplementary Information:**

The online version contains supplementary material available at 10.1186/s13053-025-00324-9.

## Background

Lynch syndrome [1] is the most common cause of inherited colorectal cancer (CRC). Other cancer types are associated with this syndrome, the most frequent being endometrial cancer in women. The syndrome is due to error in DNA mismatch repair (MMR) and is caused by (likely) pathogenic germline variants in *MLH1* (OMIM * 120,436), *MSH2* (OMIM * 609,309), *MSH6* (OMIM * 600,678), *PMS2* (OMIM * 600,259) or deletions in *EPCAM* (OMIM * 185,535). In families suspected of Lynch syndrome, tumor testing for microsatellite instability (MSI) or missing expression of MMR proteins may be performed [1]. However, direct genetic testing of DNA from blood samples is becoming more common. Many kinds of disease-causing variants are reported in Lynch syndrome, from small nucleotide changes to deletion of one or more exons (copy number variant; CNV) in one of the MMR genes. Both small nucleotide changes and CNVs can be detected by Next generation sequencing (NGS). Despite the increased use of NGS technology, we do not detect a causative variant in all families suspected to have Lynch syndrome.

Retrotransposons (RT) are repetitive sequences interspersed throughout the genome, and they have the ability to change their position (Reviewed in Bourque et al. 2018 [2]). They comprise more than 40% of the human genome, and it has been known for decades that they may cause human genetic diseases [3]. The three main types involved are long-interspersed nuclear element 1 (LINE-1, around 6 kb), short-interspersed elements (SINEs including Alu, around 300 bp) and SINE-VNTR-Alu (SVA, around 2 kb) (Reviewed in Payer and Burns 2019 [4]). The two general mechanisms RT may cause disease are 1) deletions by recombination of i.e. inverted Alu elements and 2) insertions and further disturbance of the sequence.

The focus on this paper is on Lynch syndrome caused by insertions of RT. We describe a family suspected to have Lynch syndrome where we have identified a RT insertion in *MSH6* to be the cause of their cancer predisposition. In addition, we have reviewed the literature for similar events in Lynch syndrome families.

## Case presentation

### Family description

**Fig. 1:**
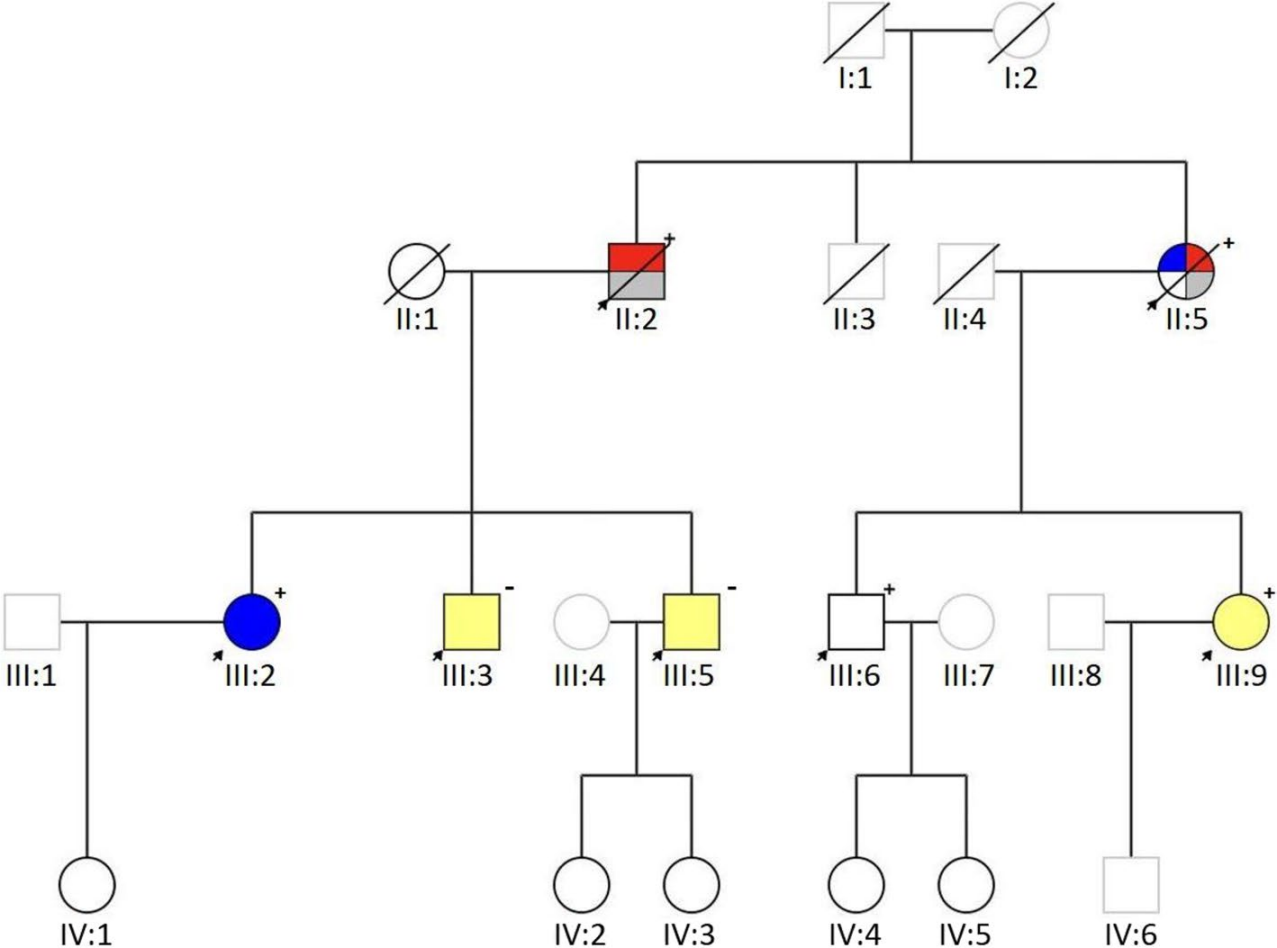
Family pedigree. The arrows indicate the family members investigated and genetic tested through our medical genetics department. The + indicates that the insertion in *MSH6* was detected, while the – indicates a normal result. The mutation carriers also shared the same haplotype. The different tumors and/or polyps in the family members are color-coded. Red: Colorectal cancer. Blue: Ovarian cancer. Grey: Urinary tract cancer. Yellow: Colon polyps. The genetic status of family members IV:1-IV:6 is currently unknown

Genetic diagnostics in this family started roughly twenty years ago. Two siblings, one male (Fig. 1, II:2) and one female (Fig. 1, II:5), were suspected to have a hereditary cancer predisposition syndrome, and Lynch syndrome was suspected. The male patient had been diagnosed with a papillary urothelial carcinoma in the bladder at 77 years of age. He was then treated for colon cancer at age 78. Finally, rectal cancer was discovered at age 84. His sister had developed several cancers at an earlier age. She was treated for ovarian cancer at age 36. Later, she was diagnosed with ureteral cancer at age 57 and then colorectal cancer at age 60. In addition, the daughter of the male patient was diagnosed with ovarian cancer at age 46.

No pathogenic variant was detected before the two siblings passed away. Family members in the generation below, who were suspected to have an increased risk of cancer development, were offered regularly cancer screening for Lynch syndrome-related tumors. There have not been any new incidents of cancer in the family members since then.

A pedigree of the family is shown in Fig. 1.

### Initial analysis did not detect a pathogenic variant, but indicated further analysis

**Fig. 2:**
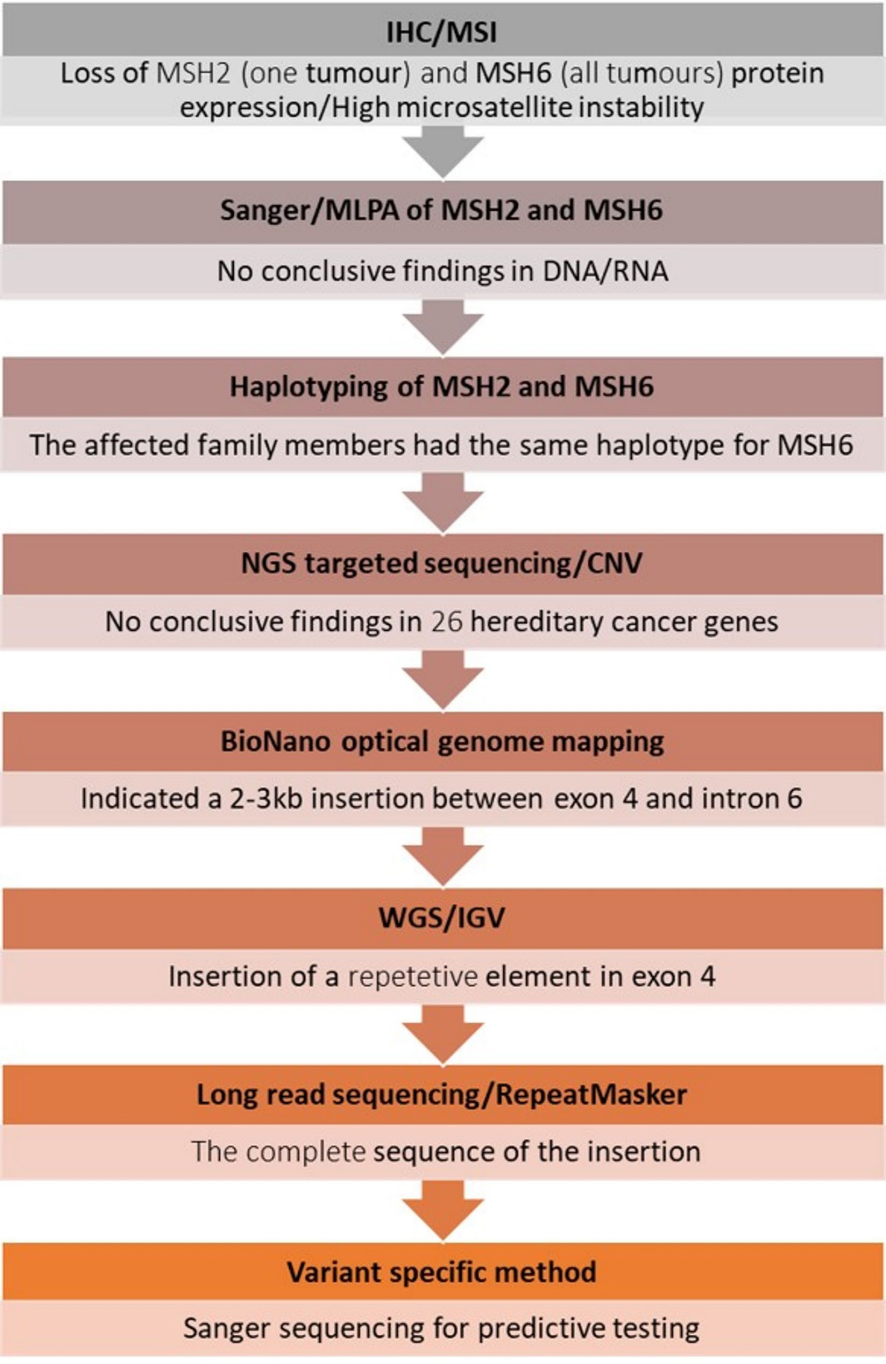
Analyses performed in one or more family members during the two decades of testing. See text for more details. IHC: immunohistochemistry, MSI: microsatellite instability, CNV: copy number variant, WGS: whole genome sequencing, IGV: Integrative Genomics Viewer

Immunohistochemistry (IHC) and MSI analyses were performed on tumors from the three affected family members. IHC showed missing protein staining for MSH2 and MSH6 in one tumor, and MSH6 in two tumors. All three tumors showed microsatellite instability. Therefore, *MSH2* and *MSH6* were analyzed by Sanger sequencing, revealing two variants (*MSH2* c.815C>T and *MSH6* c.1720T >A) in all three affected family members. These two variants were suspicious at first. However, they have later been classified as benign and likely benign, respectively. Multiplex ligation-dependent probe amplification (MLPA) analysis showed normal results; no copy number variant was detected. We further performed haplotype analysis for *MSH6* and *MSH2*, and the three affected family members (Fig. 1, II:2, II:5; III:2) had the same haplotype for nine *MSH6* markers. Two family members unaffected with cancer shared the same haplotype as the affected ones (III:6, III:9), while two unaffected (III:3 and III:5) had different haplotypes for the *MSH6* markers. Further, results from RNA analyses did not show any aberrant spliced transcript, however, there was indication of some abnormality. When using a reverse primer binding to *MSH6 *exon 4 (position c.2418_2438; NM_000179.3) the *MSH6* c.1720T >A heterozygote variant was detected. However, when using a reverse primer binding further down in exon 4 (c.3028) or in 3’UTR of the gene (Supplementary Fig. 1), only the wild type c.1720T (no A) was seen. Since MLPA analysis was normal, this cDNA result indicated that there could be a balanced structural variant in the *MSH6* gene, causing the cancer burden in the family. NGS cancer panel testing (NGS target-sequencing of selected cancer-related genes) did not uncover any pathogenic variant. Figure 2 shows a flow chart of the different analyses performed.

### Further analysis detected an RT insertion in exon 4 of MSH6

**Fig. 3:**
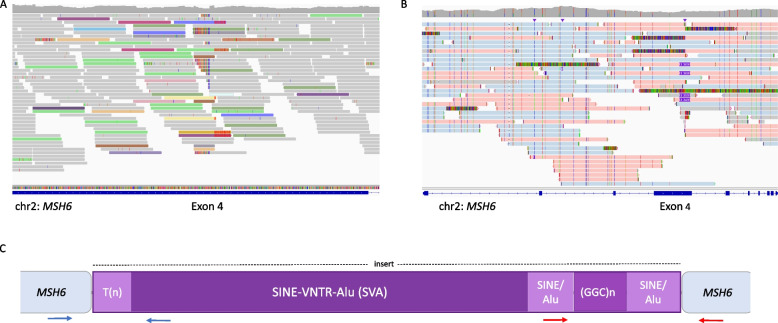
**A** Reads from WGS visualized in IGV show discordant reads in *MSH6* exon 4 mapping to other chromosomes. Light green represents reads with mate mapping in chromosome 9, darker green represents reads with mate mapping in chromosome 3. **B** Reads from LRS visualized in IGV show four reads spanning the full insertion (purple boxes) in *MSH6* exon 4. Pink represents allele 1 containing the insertion, blue represents allele 2. **C** Structure of the insertion (purple) identified by RepeatMasker. Arrows show the location of the PCR primer sets used in the variant-specific method. Blue arrows represent primer set 1 (PS1), red arrows represent primer set 2 (PS2)

As newer methods became available, we wanted to follow-up on this family as previous methods had suggested an irregularity in *MSH6*. At this time, one of the three affected family members was still alive (III:2), and fresh DNA could be isolated for new analysis using optical genome mapping (OGM; Bionano). OGM has been demonstrated to enable the detection of structural variations from a few kilobases to several megabases [5]. OGM analysis indicated a 2–3 kb insertion somewhere between exon 4 and intron 6 in *MSH6*. We therefore performed Whole Genome Sequencing (WGS) on this sample and manually inspected the mapped reads in this region using Interactive Genomics Viewer (IGV). We observed a high number of discordant reads in exon 4, where the mate mapped to other chromosomes and a breakpoint region was found (chr2:48,027,834; hg19, Fig. 3A). Most of the forward reads had their mate in a repetitive region on chromosome 9, while most of the reverse reads had their mate from the other end of the same repetitive region on chromosome 9 (2.7 kb length; inverted) or from chromosome 3, where a similar repetitive sequence was located. When looking further into this region (chr9:123,257,037–123,259,910; hg19) in the UCSC Genome Browser [6], we saw that this was a region found in many genomic sites (Supplementary Fig. 2), suggesting a common transposable element. In summary, the WGS data detected an insertion of a transposable element in exon 4 and gave the exact breakpoint regions. However, the exact length and sequence of the insert could not be identified using WGS, as this is limited by the short reads (150 bp) and short DNA fragment length (~ 450 bp). A structural variant (SV) was also detected by the SV caller in DRAGEN (Illumina) at the exact breakpoint, but no info on SV type or length was given.

To determine the exact length and sequence of the insertion, long-read sequencing (LRS) was performed. LRS identified the insertion at *MSH6* exon 4, at the expected position. LRS had 20 × coverage of the region. In total, four reads spanned the insert (Fig. 3B). In addition, five more reads spanned part of the insert. A consensus sequence was assembled from the reads spanning the insert (Supplementary Fig. 3). The total length of the insertion was estimated to be 3,379 bp. The web-based tool RepeatMasker identified most part of the insertion sequence as a SVA region with two SINE/Alu elements and two simple repeat structures (Fig. 3C) (Supplementary Table 1). In addition to the inserted RT element, nucleotides are duplicated on each side of the insert. This is called target site duplication (TSD). The HGVS nomenclature of the variant was denoted as NM_000179.3(MSH6):c.2726_2727ins[SVA[3293];2713_2726], where 2713_2726 is the TSD.

### Variant-specific PCR method

Variant-specific primer sets were designed to cover both sides of the insert, giving PCR products of 485 bp and 475 bp respectively, when the SVA insert is present. Sanger sequencing confirms the insert of the SVA element (Supplementary Fig. 4, Supplementary Table 2).

The insert was detected in the five family members sharing the same haplotype and was not detected in two family members not sharing the haplotype (Fig. 1).

### Results from literature review

We used databases and literature to search for Lynch syndrome caused by insertion of RT leading to aberrant MMR proteins (Table 1). Deletions caused by recombination of i.e. inverted Alu elements were excluded. We found ten journal papers in total, describing 15 RT insertions as cause of Lynch syndrome [7–16]. In addition, we found 24 RT insertions in the four MMR genes reported to ClinVar. Some of these variants are probably the same variant (i.e. *PMS2* c.804-64_804-63insSVA is reported four times). The nomenclature of the variants may also be misleading, as TSD may have been included in the inserted sequence. Hence, some of the variants that look like different variants, may be the same variant (i.e. *MSH6* c.3510_3511insAlu and c.3522_3523ins113 bp). Not all reports state which RT elements are inserted, and others do not describe the size of the insert. This makes it therefore impossible to compare the variants and tell if they are the same variant or not. However, what we can say is that some positions in the MMR genes seem to be more prone to RT insertions, since RTs of different sizes are inserted in the same sequence position, like *MSH6* c.3312_3313insSVA (2.4 kbp) and *MSH6* c.3330_3331ins124 bp.

**Table 1:** Literature review of retrotransposons (RT) insertions in MMR genes causing Lynch syndrome (copy number variants caused by recombination of RT are not included)

Gene	Exon	Insertion	Approx. length^a^	Year	Method	Reference (PMID and/or ClinVar)^b^
MSH2	Int 1	c.212-15_212-14insAlu	?	2022		ClinVar AG RCV002417622.2
MSH2	2	c.212-4_212-3insAlu	366 bp	2022	Long-read	36037994
MSH2	2	c.324_325insAluY	500 bp	2017	Targeted PCR	29025590
MSH2	2	c.324_325insAluY	116 bp	2024		ClinVar LG RCV003758618.3
MSH2	3	c.561_562insSVA	2.4 kbp	2020	Targeted PCR	33293698
MSH2	5	c.882_883insSVA	?	2019		ClinVar LG RCV001089791.2
MSH2	6	c.972_973insAluJ	184 bp	2004	Targeted PCR	15340835
MSH2	9	c.1442_1443insAlu	?	2022		ClinVar AG RCV002394376.2
MSH2	11	c.1746_1747insAluY	350 bp	2017	Quantitative PCR	29025590
MSH2	11	c.1746_1747ins?	102 bp	2021		ClinVar LG RCV001915515.5
MSH2	12	c. 1972_1973insSVA	3 kbp	2021	RT- and long-range -PCR	33822432
MSH2	12	c. 1972_¬1973insAlu	?	2019		ClinVar LG RCV001089843.2
MLH1	1	c.110_111insAluY	345 bp	2018	Long-range -PCR	29790873
MLH1	2	c.153_154insAlu	?	2023		ClinVar AG RCV004522867.1
MLH1	2	c.168_169ins?	151 bp	2020		ClinVar LG RCV001386567.5
MLH1	3	c.242_243insL1	1.5 kbp	2017	NGS dosage	29025590
MLH1	6	c.512_513insAlu	?	2019	NGS and cDNA	30815977
MLH1	Int 7	c.588 + 8_588 + 9insAlu	115 bp	2020	Targeted NGS	33058565
MLH1	Int 7	c.588 + 9_588 + 10ins LINE1	?	2019		ClinVar LG RCV001089854.2
MLH1	9	c.686_687insL1	6 kbp	2017	Quantitative PCR	29025590
MLH1	9	c.760_761insSVA	2 kbp	2018		ClinVar LG RCV001387732.5
MLH1	9	c.760_761ins?	145 bp	2024		ClinVar LG RCV001382366.5
MLH1	9	c.776_777ins?	138 bp	2021		ClinVar LG RCV001871284.5
MLH1	12	c.1369_1370ins?	130 bp	2020		ClinVar LG RCV001994549.5
MLH1	Int 15	c.1667 + 678insAlu	?	2022		ClinVar AG RCV002403904.2
MLH1	18	c.2004_2005insAlu	?	2024		ClinVar AG RCV004522881.1
MSH6	4	c.1826_1827insAlu		2022		ClinVar AG RCV003182628.2
MSH6	5	c.3311_3312insSVA		2022		ClinVar AG RCV002326319.2
MSH6	5	c.3312_3313insSVA	2.4 kbp	2020	Targeted PCR	33293698
MSH6	5	c.3330_3331ins?	124 bp	2022		ClinVar LG RCV001892249.5
MSH6	6	c.3510_3511insAlu		2023		ClinVar AG RCV002459211.2
MSH6	6	c.3522_3523ins?	113 bp	2023		ClinVar LG RCV003594765.3
PMS2	Int 7	c.804-64_804-63insSVA		2019		ClinVar AG RCV002412441.2
PMS2	Int 7	c.804-60_804-59insSVA_F	2.2 kbp	2012	Southern blot	22461402
PMS2	Int 7	c.804-60_804-59insSVA_F	2.2 kbp	2017	NGS dosage	29025590
PMS2	Int 7	c.804–60_804-59insSVA		2023	Transcript capture and long-read RNA sequencing	36593122 ClinVar AG RCV002412440.2
PMS2	11	c.1273_1274ins?	115 bp	2021		ClinVar LG RCV001941567.6
PMS2	11	c.1274_1275insALU		2020		ClinVar AG RCV002378917.2
PMS2	11	c.1848_1849insAlu		2019		ClinVar LG RCV001089839.2

^a^Approximate length included if information available

^b^PMID: PubMed reference number. Laboratories reported to ClinVar are abbreviated as AG: Ambry Genetics; LG: Labcorp Genetics (formerly Invitae). The ClinVar accession numbers are given (RCV)

The first report we found was from 2004, where an Alu insertion of less than 200 bp in *MSH2* exon 6 caused Lynch syndrome [10]. The second report was from 2012, reporting a SVA of 2.2 kbp inserted in *PMS2* intron 7 [15]. The next five reports are from 2017, and after 2017, two to seven RT of different sizes have been reported each year (Table 1). Short RTs (< 500 bp) like Alus were most common (26 variants), followed by SVAs of 2–3 kbp (10 variants), and less common were LINE/L1 RTs (three variants).

## Discussion and conclusions

The family described in this case report has been followed for two decades by our Genetic department, and Lynch syndrome has been the suspected diagnosis. After 20 years, we are finally able to confirm Lynch syndrome at the molecular level, as we have identified one SVA insertion of > 3 kbp in exon 4 of *MSH6*. We can now offer predictive testing to the family with the variant-specific Sanger sequencing method that we designed to confirm the SVA insert.

As shown in Fig. 1, several family members did develop benign colon polyps at some point, but only family members with the SVA insertion in *MSH6* developed cancer. However, not all family members with the insertion have developed cancer. The family history is in accordance with the reduced and age-related disease penetrance of Lynch syndrome with a pathogenic alteration in *MSH6* [17].

The literature review was performed to decide whether RT insertions are a common cause of Lynch syndrome. We found in total around 40 variants, 15 in 10 journal papers [7–16] and the rest in ClinVar. Some of them are probably the same variant. Pathogenic and likely pathogenic variants in MMR genes in ClinVar include 7500 different variants (ClinVar February 2025), 2334, 2137, 1982 and 1049 in *MSH2*, *MSH6, MLH1* and *PMS2* respectively. RT insertions causing Lynch syndrome reported per this date make then up about 0.5% (40 out of 7500). Thus, RTs insertions are not a common cause of Lynch syndrome, but the number might be underestimated because of the difficulties in detecting these variants with Sanger sequencing and NGS targeted sequencing.

The medical follow-up and diagnostic methods applied to detect the genetic variant leading to cancer predisposition in this family, also reflect the technological advancement in methods for detecting DNA variants for the past 20 years. At the start of this diagnostic journey, Sanger sequencing was the gold standard. The low throughput and labor-intensive nature of this method enabled analysis of only a few suspected genes. Although MSI/IHC results indicated Lynch syndrome, Sanger sequencing has limitations in detecting RT, so no pathogenic variant could be identified. Haplotype analysis showed that the affected family members had the same haplotype for *MSH6*. Later, NGS technology with higher throughput was introduced and adopted in diagnostics at our lab. Presently, hereditary cancer cases are routinely analyzed on a NGS targeted sequencing panel, where cancer-relevant genes are sequenced in parallel. Although we detect CNVs by this method, in addition to single nucleotide variants (SNV) and small insertions/deletions (InDels), we do not have callers for other SVs, like larger insertions. However, when visually inspecting our NGS target sequencing short-read data in retrospect, we did observe many discordant reads, both split reads and reads with clipped bases, at the breakpoint region in exon 4. The sample was further analyzed using OGM, and the results indicated a 2–3 kb insertion somewhere between exon 4 and intron 6. OGM is a novel method capable of detecting complex SVs [18], but are not able to accurately pinpoint SV breakpoints as it lacks sequence-level resolution [19]. We recently also established genome sequencing in our diagnostic lab which is a more comprehensive analysis compared to targeted NGS methods as it provides more uniform coverage across both coding and non-coding regions enabling better detection of CNVs. When performing CNV and SV calling on the WGS data, an SV was called at the exact position. When visualizing the WGS data in IGV, we could in addition to the exact breakpoints, also see the start and end sequence of the insert. As expected, there were no CNV detected by the CNV caller.

LRS is increasingly being used in diagnostics due to its ability to enhance diagnostic yield by providing comprehensive genomic insights [20, 21]. In our lab, we are currently testing the use of Oxford Nanopore Technology in some challenging clinical cases. In this family, the LRS analysis provided the exact length and sequence of the full insertion, which were not possible from WGS data due to the short DNA fragments and short read length.

NGS targeted sequencing is often the first-tier test in routine diagnostics of hereditary cancer [22, 23]. Usually, this technology is limited to SNV/InDels and sometimes CNVs. Although newer bioinformatic tools might be capable of detecting SVs in targeted sequencing data [24, 25], this remains challenging due to the uneven coverage and the limitation to the sequenced regions, which is primarily exons. As a result, insertions in non-coding regions are likely missed.

LRS can generate long reads that span entire retrotransposons, allowing for the identification of full-length insertions, which is not possible with short reads. Long reads excel in mapping complex genomic regions, including those rich in retrotransposons, where short reads might struggle due to repetitive sequences and structural complexity. In cases where there are indications of irregularities in specific regions of the genome, long-read technology is an excellent second-tier test to help resolve complex variants.

RNAseq (NGS used to analyze cDNA) is one approach that has been shown in previous studies to be well suited to detect pathogenic variants in MMR genes, especially for deep intronic variants causing aberrant splicing [16, 26]. This method is not currently in clinical use in our diagnostic laboratory, as we use Sanger sequencing of cDNA for the four MMR genes. However, this is also a highly relevant method, but will also need to be followed up with other analysis like WGS or LRS to detect the specific genetic cause if aberrations are found.

Ideally, a single method capable of detecting all pathogenic variants would be the absolute preference. However, this is currently not feasible. The optimal workflow for analyzing samples from patients suspected of Lynch Syndrome depends heavily on the laboratory’s available equipment, analytical capabilities, and cost structure. Cost per analysis can vary significantly depending on the sequencing platform and sample throughput. For example, the cost of WGS may differ based on the specific sequencing platform used and the number of samples processed. Generally, larger platforms with higher throughput offer lower per-sample costs. Consequently, high-capacity sequencing laboratories may be able to perform WGS at a cost comparable to targeted sequencing in smaller labs. Testing strategies must therefore balance cost-effectiveness with diagnostic yield.

If costs were not a limiting factor, the ideal first-tier test would be WGS combined with a virtual cancer panel. This approach enables comprehensive variant detection, including precise breakpoint identification and characterization of insert sequences. Subsequent analyses would be guided by the findings from WGS.

LRS is particularly valuable as a second-tier test when WGS reveals SVs requiring further resolution, as demonstrated in the case presented here. Based on our experience with the Oxford Nanopore system, LRS still faces challenges in InDel detection accuracy and in the performance of bioinformatic SV callers, especially in filtering clinically relevant variants. Nonetheless, these technologies are rapidly advancing and are becoming increasingly relevant for routine clinical use.

The literature review performed on inserted elements in the MMR mismatch repair genes, suggests that these insertions have been detected mostly using PCR-based methods. With the introduction of newer methods as described above, we expect more of these cases to be solved, and in a much shorter time. This will give a more accurate picture of how commonly retrotransposons cause Lynch syndrome and other genetic diseases.

## Methods

### Molecular genetic analyses performed in the family

Genetic examination of the family started nearly 20 years ago. The standard testing at that time was to do IHC of the four MMR proteins MLH1, MSH2, MSH6 and PMS2 and MSI testing in tumor material. If the tumor analyses showed missing protein staining and MSI, Sanger sequencing and MLPA (MRC-Holland) were performed for the genes who were indicated to be aberrant out from IHC results. From 2012, gene panel testing by NGS was done, first by pyrosequencing (GS Junior from Roche; four MMR genes), and from 2017 by Illumina sequencing technology (Applied Biosystems, 26 hereditary cancer genes including the four MMR genes). To look for intronic variants causing aberrant splicing, RNA analyses (cDNA) were performed. All these methods have been described before [27–29].

### Haplotyping

Haplotype analysis was performed to assess indications of a hereditary germline mutation in the family. We investigated whether affected family members shared a common haplotype for one of the MMR genes. IHC analyses indicated that MSH2 and/or MSH6 protein was not expressed. The haplotyping was performed by analyzing a set of seven microsatellite markers and 13 different single nucleotide polymorphisms (SNPs) distributed over the *MSH2* and *MSH6* genes, both located on chromosome 2p14-2p21. The length of the microsatellite markers was found by fragment analyses on ABI3500, and the SNPs were analyzed by Sanger sequencing. The primers for microsatellite markers and SNPs may be obtained by request.

### Optical genome mapping (OGM) analysis

OGM analysis (Bionano) was performed as described previously [18]. Peripheral blood samples were frozen at − 80 ◦C within 4 days after withdrawal, and ultra-high molecular weight DNA (UHMW DNA) from approximately 1.5 × 10^*6*^ cells was extracted using “SP Blood & Cell Culture DNA Isolation Kit” (Bionano Genomics, San Diego, CA, USA). UHMW DNA was labeled using the DLS DNA Labeling Kit (Bionano) according to the manufacturer’s instructions. The labeled DNA was applied to G1.2 flow cells and analyzed on a Saphyr instrument (Bionano), following Saphyr System User Guide revision D. The Saphyr chip was run targeting 320 Gb data per sample (100X coverage), yielding 500 Gb data. After default filtering, mean molecule length was 251 kb, median 224 kb and N50 was 255 kb. The virtual DNA strands were de novo assembled, and the consensus maps of the molecules were aligned to the human reference genome (GRCh37) and visualized in Bionano Access software (version 1.7). Recommended filtering was used corresponding to the following minimum confidence values: insertion/deletion = 0, inversion = 0.7, duplications = −1, intra-fusion/inter-translocation = 0.05, CNV = 0.99, and aneuploidy = 0.95. Events detected were subsequently filtered against a normal samples database and only variants absent (or present at a percentage below 1%) of that database were considered for analysis.

### Whole genome sequencing

Total genomic DNA was isolated from EDTA peripheral blood using the QIAsymphony DNA MIDI kit (Qiagen). DNA concentration was measured using the Spark microplate reader (Tecan, Switzerland). Sequencing libraries were prepared using the Illumina DNA PCR-Free Prep kit (Illumina, Catalog#: 20,041,794) with 500 ng DNA input, and sequenced on an Illumina NovaSeq6000 using 2 × 150 bp reads. The sample yielded a medium coverage of 36x. Illumina DRAGEN pipeline (version 4.2.4) [30] was used for bioinformatics data analysis.

### Long-read sequencing

A.bed file was created that included a total of 0.108% of the human genome, including the *MSH6* gene. The coordinates for the genes were adapted from Gencode Basic gene annotation Release 38 for hg38. In addition, an extra 20 kb was added on each side of the coordinates as buffer regions. Total genomic DNA was isolated as described for whole genome sequencing. Prior to sequencing, 2 µg of genomic DNA was sheared to 10 kb using a Covaris g-TUBE (Covaris, 520,079), following the manufacturer’s instructions. The sheared DNA was analyzed on an Agilent 4200 TapeStation using Genomic DNA reagents (Agilent, 5067–5366) and Genomic DNA ScreenTape (Agilent, 5067–5365). DNA concentration was measured on a Qubit 3.0 fluorometer using the Qubit dsDNA HS Assay kit (Invitrogen, Q32851).

The library for sequencing was prepared using the SQK-LSK110 Ligation Kit (Oxford Nanopore Technologies, ONT) according to the manufacturer’s instructions. The library was loaded on an R10.4.1. flow cell (ONT, FLO-MIN114) for sequencing on a MIN-101B MinION instrument running the MinKNOW version 23.04.6. Sequencing was performed in a fast basecalling mode by Guppy version 6.5.7 along with the enriched adaptive sampling option up to 72 h. An additional library loading at 24 h was performed after nuclease flushing of the flow cell using the Flow Cell Wash Kit (ONT, EXP-WSH004).

### Bioinformatic analyses long-read sequencing

The raw signals (.pod5 files) from the sequencing run were subjected to Super accurate basecalling using the dna_r10.4_1_e8.2_400bps_sup.cfg model within MinKnow. Nanoplot version 1.41.6 was used for quality control of the reads. The basecalled reads were aligned to the hg38 reference genome using Minimap2 version 2.26. The resulting BAM file was visualized in IGV. Qualimap version 2.2.2-dev was used for mapping quality metrics of the BAM file. To achieve a consensus on the insertion element, the reads aligning to *MSH6* were extracted using samtools and assembled into contigs using canu Version 2.2. The consensus alignment of the insertion was analyzed for transposable elements using the web-based tool RepeatMasker (https://www.repeatmasker.org/cgi-bin/WEBRepeatMasker).

### Variant-specific method (Sanger-sequencing)

SVA insert-specific PCR primer sets were designed, one that covered the 5’ end of the insert (Primer set; PS1) and one that covered the 3’ end of the insert (Primer set 2; PS2, Fig. 3C). PCR amplification was performed using 1X AmpliTaq Gold™360 DNA polymerase (Applied Biosystems), 360 GC Enhancer (Applied Biosystems), 30 ng DNA template, and 0.2 µM of each primer. PCR annealing with PS1 primers was performed at 60° C for 40 cycles, and for PS2 a touchdown approach at 68–60°C was applied for 16 + 24 cycles. The presence of the SVA insert resulted in a product of 485 bp with the PS1 primers and 475 bp with the PS2 primers (Supplementary Fig. 4). Sanger sequencing of purified PCR products was performed with Big Dye™ Terminator v3.1 Cycle Sequencing Kit (Applied Biosystems) using standard conditions, and the purified Cycle sequencing products were run on ABI3730 (Applied Biosystems). The sequencing results were analyzed using Sequence Scanner 2 (Applied Biosystems). PCR with both the primer sets mentioned above was performed for all the members in the family, and when the SVA insert was present, the PCR product was sequenced by Sanger sequencing to confirm the sequence identity. A duplex PCR containing an internal control was also applied to all samples from the family to ensure there were no false negative amplifications.

### Search parameters for literature review

Review of the literature was performed by searching for combinations of Lynch syndrome, Mismatch repair, HNPCC, insertion of transposable elements, Alu, SVA, LINE. Databases searched included PubMed (https://pubmed.ncbi.nlm.nih.gov/), ClinVar (https://www.ncbi.nlm.nih.gov/clinvar/) and Google scholar (https://scholar.google.com/).

## Supplementary Information

Supplementary Material 1

Supplementary Material 2

## Data Availability

Because of national data protection rules and privacy concerns raw sequencing data will not be publicly available. Data may be shared upon request.

## Abbreviations

CNV: Copy number variant
CRC: Colorectal cancer
IGV: Interactive Genomics Viewer
IHC: Immunohistochemistry
InDels: Insertions/deletions
LINE-1: Long-interspersed nuclear element 1
LRS: Long-read sequencing
MSI: Microsatellite instability
MLPA: Multiplex ligation-dependent probe amplification
MMR: Mismatch repair
NGS: Next generation sequencing
OGM: Optical genome mapping
RT: Retrotransposon
SINEs: Short-interspersed elements
SNP: Single nucleotide polymorphism
SNV: Single nucleotide variant
SV: Structural variant
SVA: SINE-VNTR-Alu
TSD: Target site duplication
WGS: Whole Genome Sequencing
