# Supplementary material for: Lynch syndrome caused by SINE-VNTR-Alu-F retrotransposon insert in MSH6 confirmed after 20 years of testing: a case report and literature review
Source: Hered Cancer Clin Pract. 2025 Oct 14;23:22. doi: 10.1186/s13053-025-00324-9 (PMC12523002; doi:10.1186/s13053-025-00324-9)
Supplement: Supplementary file 1 — Supplementary Material 1 [file 13053_2025_324_MOESM1_ESM.pdf]

Supplementary Figures to the article: Lynch syndrome caused by SINE-VNTR-Alu-F retrotransposon insert in MSH-6 confirmed after 20 years of testing: a case report and literature review.

Supplementary Figure 1

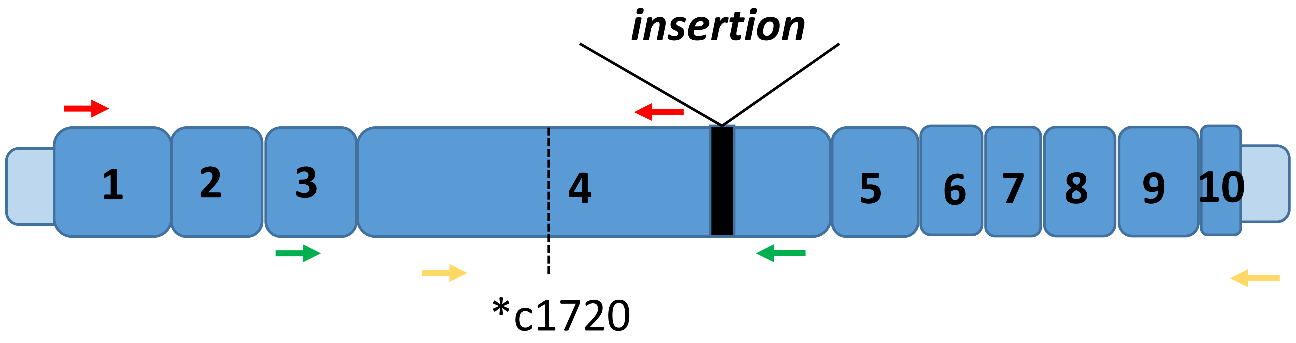

Primer	Localization	Coding position (NM_000179.3)	Sequence (5 -> 3)
Red-forward	Exon 1	c.3-c.21	GTCGCGACAGAGCACCCTG
Red-reverse	Exon 4	c.2442-c.2461	GCCTCTCAAGATCTGGAAGC
Green-forward	Exon 3	c.466-c.484	TCAAAGGAAGCCCAGAAGG
Green-reverse	Exon 4	c.3028-c.3048	AGCCAACTTCTTTTCAATAGT
Yellow-forward	Exon 4	c.1267-c.1287	CTTGTCATCTGTTACAAGGTG
Yellow-reverse	3'UTR	c.*21-c.*42	ACCTTTGTCAGAAGTCAACTCA

Supplementary Figure 1.
The sketch shows the MSH6 gene, exons marked 1-10. RT-PCR identified a heterozygous T>A mutation in position c.1720, but only when using the primer pair marked in red. The 2 other primer pairs (green, yellow), did not show this mutation. This suggested a structural variant disturbing the sequence of one of the alleles, which we later showed to be an insertion. Primer pairs sequence and localisation is shown in the box to the right.

Supplementary Figure 2

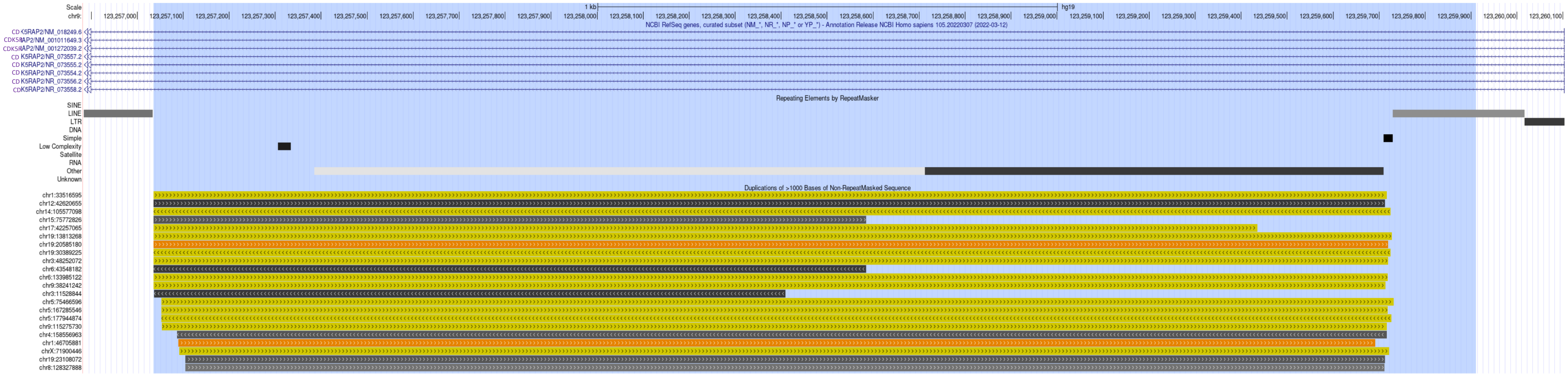

Supplementary Figure 2. Analysis of the region in chromosome 9 (chr9:123,257,037-123,259,910; hg19) using the UCSC Genome Browser, suggests that this is a genomic sequence located at many different sites in the genome.

Supplementary Figure 3

TTGACTGTAGAATTTTTTTTTTTTTTTTTTTTTTTTTTTTTTTTTTTAAAATTTATTTTTTTATTGATAATTCTTGGGTGTTTCTCACAGAGG
GGGATTTGGCAGGGTCATGGGACAATAGTGGAGGGAAGGTCAGCAGATAAACAAGTGAACAAAGGTCTCTGGTTTTCCTA
GGCAGAGGACCCTGCGGCCTTCCGCAGTGTTTGTGTCCCTGATTACTTGAGATTAGGGATTGGTGATGACTCTTAACGAGC
ATGCTGCCTTCAAGCATCTGTTTAACAAAGCACATCTTGCACCGCCCTTAATCCATTTAACCCTGAGTGGACACAGCACATG
TTTCAGAGAGCACAGGGTTGGGGGTAAGGTCACAGATCAACAGGATCCCAAGGCAGAGGAATTTTTCTTAGTGCAGAACA
AAATGAAAAGTCTCCCATGTCTACTTCTTTCTACACAGACACGGCAACCATCCGATTTCTCAATCTTTTCCCCACCTTTCCCG
CCTTTCTATTCCACAAAGCCGCCATTGTCATCCTGGCCCGTTCTCAATGAGCTGTTGGGCACACCTCCCAGACGGGGTGG
TGGCCGGGCAGAGGGGCTCCTCACTTCCCAGTAGGGGCGGCCGGGCAGAGGCGCCCCTCACCTCCCGGACGGGGC
GGCTGGCCGGGCAGGGGGGCTGACCCCCCCACCTCCCTCCTGGACGGGGCGGCTGGCCGGGCGGGGGGCTGACC
CCCCAACCTCCCTCCCGGATGGGGCGGCTGGCCGGGCGGGGGGCTGACCCCCCCACCTCCCTCCCGGACGGGGC
GGCTGGCCGGGCAGAGGGGCTCCTCACTTCCCAGTAGGGGCGGCCGGGCAGAGGCGCCCCTCACCTCCCGGACGG
GGCGGCTGGCCGGACGGGGCGGCTGGCCGGACGGGGGGCCGACCCCCCCACCTCCCTCCCGGACGGGGCGGCT
GGCCGGGCGGGGGGCCGACCCCCCCACCTCCCTCCCGGACGGGGCGGCTGGCCGGGCGGGGGGCCGACCCCCC
CACCTCCCTCCCGGACGGGGCGGCTGGCCGGGCAGAGGGGCTCCTCACTTCCCAGTAGGGGCGGCCGGGCAGAGG
CGCCCCTCACCTCCCGGACAGGGCGGCTGGCTGGGCGGGGGGGCTGACCCCCCCCCACCTCCCTCCCGGACGGG
GCGGCTGGCCGGGCGGGGGGCTGACCCCCCCACCTCCCTCCCGGACGGGGCGGCTGGCCGGGCAGAGGGGCTCC
TCACTTCCCAGTAGGGGCGGCCGAGCAGAGGCGCCCCTCACCTCCCGGACGGGGCGGCTGGCCGGGCAGGGGGGC
CGACCCCCCCCCCCACCTCCCTCCCGGACAGGGCGGCTGGCCGGGCGGGGGGCCGACCCCCCCACCTCCCTCCC
GGACGGGGCGGCTGGCCGGGCGGGGGGCCGACCCCCCCACCTCCCTCCCGGACAGGGCGGCTGGCCGGGCAGA
GGGGCTCCTCACTTCCCAGTAGGGGCGGCCGGGCAGAGGCGCCCCTCACCTCCCGGACAGGGCGGCTGGCTGGGC
GGGGGGGCTGACCCCCCCCAACCTCCCTCCCAGACGGGGCGGCTGGCCGGGCGGGGGGCTGACCCCCCCACCTC
CCTCCCGGACGGGGCGGCTGGCCGGGCAGAGGGGCTCCTCACTTCCCAGTAGGGGCGGCCGGGCAGAGGCGCCCC
TCACCTCCCGGACGGGGCGGCTGGCCGGGCAGGGGGGCCGACCCCCCCACCTCCCTCCCGGACGGGGCGGCTGG
CCGGGCAGAGGGGCTCCTCACTTCCCAGTAGGGGCGGCCGGGCAGAGGCGCCCCTCACCTCCCAGACGGGGCGGC
TGGCCGGGCGGAGGGCTGACCCCCCCACCTCCCTCCCCGACAGGGCGGCTGGCCGGGCGGGGGGCTGACCCCCC
CACCTCCCTCCCGGACGGGGCGGCTGGCCGGGCAGAGGGGCTCCTCACTTCCCAGTAGGGGCGGCTGGGCAGAGG
CGCCCCTCACCTCCCAGACGGGGCGGCTGGCCGGGCGGAGGGCTGACCCCCCCACCTCCCTCCCGGACGGGGCG
GCTGGCCAGGCGGGGGGCTGACCCCCCTACCTCCCTACCGGACGGGGCGGCTGGCCGGGTGGGGGGGCTGACCCC
CCCATCTCCCTCCCGGACGGGGTGGCTGGCCGGGCTGAGGGGCTCCTCACTTCCCAGTAGGGGCGGCCGGGCAGAG
GCGCCCCTCACCTCCCGGACGGGGCGGCTGGCCGGGCGGGGGGCTGACCCCCCCACCTCCCTCCCGGATGGCAC
GGCTGGCCAGGCGGGGGGCTGACCCCCCCACCTCCCTCCCGGATGGCACGGCTGGCCGGGCGGGGGGGCTGACC
CCCACCTCCCTCCCGGATGGGGCGGCTGGCCGGGCGGGGGCTGACCCCCCCCCCCCACCTCCCTCCTGGACGGGG
TGGCTGCCGGGCGGAGACGCTCCTCACTTCCCAGATGGGGTGGCTGCCGGGCGGAGAGGCTCCTCACTTCTCAGACG
GGGCAGCTGCCGGGCGGAGGGGCTCCTCACTTCTCAGACGGGGTGGTTGCCAGGCAGAGGGTCTCCTCACTTCTCAGA
CGGGGCGGCCGGGCAGAGACGCTCCTCACCTCCCAGACGGGGTCTCGGCCGGGCAGAGGCGCTCCTCACATCCCA
GATGGGGCGGCGGGGCAGAGGCGCTCCCCACATCTCAGACGATGGGCGGCCGGGCAGAGACGCTCCTCACTTCCTA
GATGTGATGGCGGCTGGGAAGAGGCGCTCCTCACTTCCTAGATGGGATGGCGGCCGGGCGGAGACGCTCCTCACTTTC
CAGACTGGGCAGCCAGGCAGAGGGGCTCCTCACATCCCAGACGATGGGCGGCCAGGCAGAGACACTCCTCACTTCCC
AGACGGGGTGGCAGCCGGGCAGAGGCTGCAATCTCGGCACTTTGGGAGGCCAAGGCAGGCGGCTGCTCCTTGCCCTC
GGGCCCCGCGGGGCCCGTCCGCTCCTCCAGCCGCTGCCTCCCGGGCGGCGCTCGCCGGCGCGGCGGCAAAGACT
GAGACAGCTCCGCTGCCCGCTGAACTCCATCCTCCTGGCGGTCGGGCGGCGGCGGCTGCGGTCGGTCGCGGCAGCG
GCTCCGCTTCATATCTGCAGCTGGGGCCCGCGGGCGTCAGCGCCGCCGCGCCAACCACCAGCCGCGGCCACCATGG
CCAGACGGGCTCCCTAAGCCACCGACCCCAGCCCGCGGCGCCTTCGACCCTTCTGGGGCCTCCGGCGCCGCGACCT
CCTCTGCCTGAAATTTCTTTTTTCTTTTCCTTTTATTTTATTTTATTTTTTGAGACGGAGTCTTGCTCTGTTGTCTGGGTGGAGTGC
AGTGGTGCAATCTC

Supplementary Figure 3. Consensus sequence for the insertion.

Supplementary Figure 4

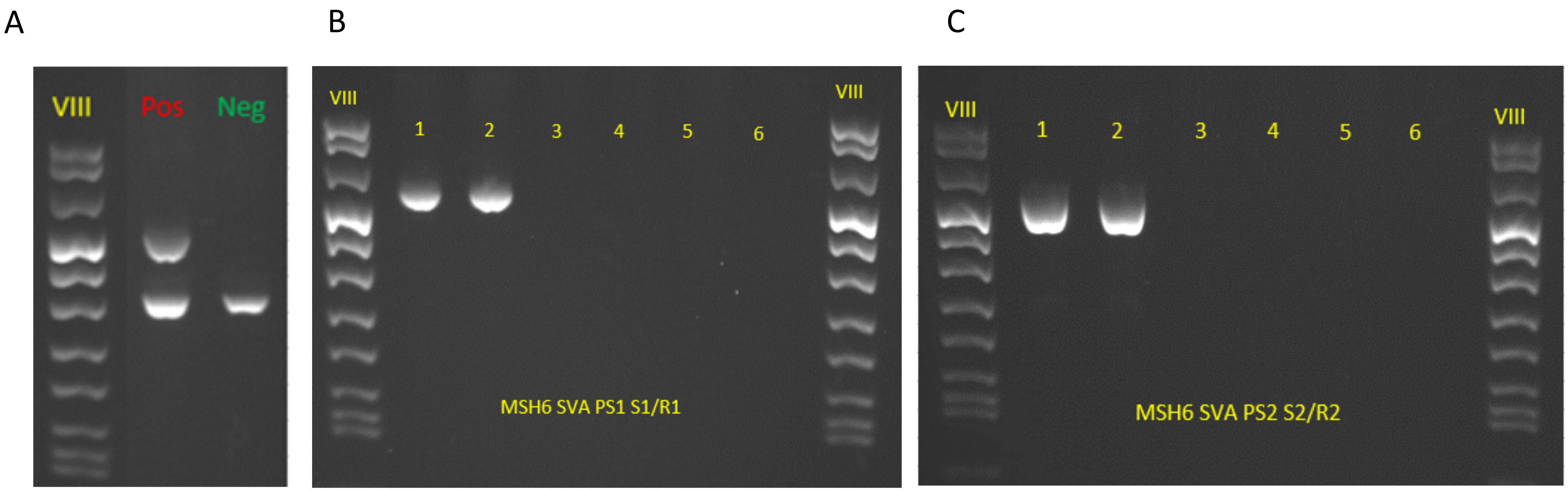

Supplementary Figure 4. Agarose gel results of the variant-specific PCR method. A) The product at 485 bp is the SVA spesific product with the PS1 primers. The product at 318 bp is an internal amplification control (a segment of MSH6-exon 4), from the control primerset. B) The product at 485 bp is the SVA spesific product with the PS1 primers. C) The product at 475 bp is the SVA spesific product with the PS2 primers. Ladder: DNA Molecular weight marker VIII.
