# Supplementary material for: Lynch syndrome caused by SINE-VNTR-Alu-F retrotransposon insert in MSH6 confirmed after 20 years of testing: a case report and literature review
Source: Hered Cancer Clin Pract. 2025 Oct 14;23:22. doi: 10.1186/s13053-025-00324-9 (PMC12523002; doi:10.1186/s13053-025-00324-9)
Supplement: Supplementary file 2 — Supplementary Material 2 [file 13053_2025_324_MOESM2_ESM.pdf]

Supplementary Tables for the article: Lynch syndrome caused by SINE-VNTR-Alu-F retrotransposon insert in MSH6 confirmed after 20 years of testing: a case report and literature review

Supplementary table 1

Output from Repeatmasker (RepeatMasker Web Server) on the insertion consensus sequence, with an added column of length of the different repeat classes.

Repeat class	Matching repeat	start	end	Size (bp)	% divergence	% deletion	% insertion
Simple repeat	(T)n	13	47	34	0.0	0.0	0.0
Retrotransposon/ SVA	SVA_F	53	2924	2871	4.1	0.2	20.9
Alu	SINE/Alu	2925	2961	36	16.2	0.0	0.0
Simple repeat	(GGC)n	3087	3132	45	12	0.0	7.7
AluSc5	SINE/Alu	3311	3379	68	14.5	0.0	0.0

Supplementary table 2

Primer sequences for the variant-specific PCR method.

Primer set	Primer name	Primer sequence* (5'→3')	Position NM_000179.3(MSH6)
PS1	MSH6-SVA-F1	*CACGACGTTGTAAAACGAC*-ATGAAGAAACTACATACAGCAAG	c.2534
PS1	MSH6-SVA-R1	*CAGGAAACAGCTATGACC*-TAAATGGATTAAGGGCGGTG	Insert-specific position
PS2	MSH6-SVA-F2	*CACGACGTTGTAAAACGAC*-TGCCCGCTGAACTCCATC	Insert-specific position
PS2	MSH6-SVA-R2	*CAGGAAACAGCTATGACC*-TGTTTCTCTAGGTATTCCAGGAGG	c.2873
Control	MSH6-ex4intctr-F	*CACGACGTTGTAAAACGAC*-CACATATATCCAAGTATGATAGAG	c.1499
Control	MSH6-ex4intctr-R	*CAGGAAACAGCTATGACC*-AGTTTCCTTTGAGAGATTTCC	c.1815

*All primers contain universal tail sequence, given in italics (*CACGACGTTGTAAAACGAC fwd, CAGGAAACAGCTATGACC rev)*.
